# Beta-Amyloid and Its Asp7 Isoform: Morphological and Aggregation Properties and Effects of Intracerebroventricular Administration

**DOI:** 10.3390/brainsci14101042

**Published:** 2024-10-21

**Authors:** Valeriya Ushakova, Yana Zorkina, Olga Abramova, Regina Kuanaeva, Evgeny Barykin, Alexander Vaneev, Roman Timoshenko, Peter Gorelkin, Alexander Erofeev, Eugene Zubkov, Marat Valikhov, Olga Gurina, Vladimir Mitkevich, Vladimir Chekhonin, Anna Morozova

**Affiliations:** 1 Department of Basic and Applied Neurobiology, V.P. Serbsky National Medical Research Center of Psychiatry and Narcology, The Ministry of Health of the Russian Federation, 119034 Moscow, Russia; zorkina.ya@serbsky.ru (Y.Z.); abramova1128@gmail.com (O.A.); zubkov.e@serbsky.ru (E.Z.); marat.valikhov@gmail.com (M.V.); hakurate77@gmail.com (A.M.); 2 Department of Higher Nervous Function, Lomonosov Moscow State University, 119991 Moscow, Russia; 3 Laboratory of Biophysics, National University of Science and Technology “MISIS”, 119049 Moscow, Russia; regina.kuanaeva@mail.ru (R.K.); vaneev.aleksandr@gmail.com (A.V.); timoshenko.rv@misis.ru (R.T.); gorelkin.pv@misis.ru (P.G.); erofeev@polly.phys.msu.ru (A.E.); 4 Engelhardt Institute of Molecular Biology, Russian Academy of Sciences, 119334 Moscow, Russia; eugenebarykin@gmail.com (E.B.);; 5 Chemistry Department, Lomonosov Moscow State University, 119991 Moscow, Russia; 6 Department of Medical Nanobiotechnology, N. I. Pirogov Russian National Research Medical University, the Ministry of Health of the Russian Federation, 117513 Moscow, Russia

**Keywords:** Alzheimer’s disease, amyloid, AFM, ROS, rat model, intracerebroventricular injection

## Abstract

Background/Objectives: One of the hallmarks of Alzheimer’s disease (AD) is the accumulation of aggregated beta-amyloid (Aβ) protein in the form of senile plaques within brain tissue. Senile plaques contain various post-translational modifications of Aβ, including prevalent isomerization of Asp7 residue. The Asp7 isomer has been shown to exhibit increased neurotoxicity and induce amyloidogenesis in brain tissue of transgenic mice. The toxicity of Aβ peptides may be partly mediated by their structure and morphology. In this respect, in this study we analyzed the structural and aggregation characteristics of the Asp7 isoform of Aβ_42_ and compared them to those of synthetic Aβ_42_. We also investigated the effects of intracerebroventricular (i.c.v.) administration of these peptides, a method often used to induce AD-like symptoms in rodent models. Methods: Atomic force microscopy (AFM) was conducted to compare the morphological and aggregation properties of Aβ_42_ and Asp7 iso-Aβ_42_. The effects of i.c.v. stereotaxic administration of the proteins were assessed via behavioral analysis and reactive oxygen species (ROS) estimation in vivo using a scanning ion-conductance microscope with a confocal module. Results: AFM measurements revealed structural differences between the two peptides, most notably in their soluble toxic oligomeric forms. The i.c.v. administration of Asp7 iso-Aβ_42_ induced spatial memory deficits in rats and elevated oxidative stress levels in vivo, suggesting a potential of ROS in the pathogenic mechanism of the peptide. Conclusions: The findings support the further investigation of Asp7 iso-Aβ_42_ in translational research on AD and suggest its involvement in neurodegenerative processes.

## 1. Introduction

Alzheimer’s disease (AD) is the most prevalent form of dementia. It affects a significant proportion of the elderly population worldwide, leading to cognitive and functional impairments and significantly reducing the quality of life for patients and their families. According to the World Health Organization, more than 55 million people suffer from dementia, and this number is expected to rise significantly by 2050 due to increasing life expectancy [1,2]. One of the challenges in treating AD is the lack of effective treatments, with current therapies primarily providing symptomatic relief [3].

To study the mechanisms underlying the disease and find new therapeutic targets, translational animal models such as rodents and non-human primates are used [4]. Due to the significant development of instrumental methods of molecular biology and genetics, AD transgenic models have become widespread. These models are created by introducing genetic variations associated with AD into the animal genome, such as mutations in amyloid precursor protein (APP), presenilin 1 (PSEN1), and presenilin 2 (PSEN2), among others [5,6]. While these models are useful for understanding the disease’s development, they only account for about 1% of cases associated with early-onset and hereditary forms of AD [7,8]. Natural aging models in non-human primates [9,10], as well as direct injections of biological and chemical substances into the brains of experimental animals [11], can be used to investigate the sporadic forms of AD. These methods allow researchers to replicate some features of neurodegenerative processes, such as behavioral changes and structural and neurochemical alterations [12,13]. Beta-amyloid (Aβ) stereotaxic injections are often used to trigger pathological disorders in animals, leading to neuroinflammation, memory impairment, and neuronal degeneration [11,14].

The basis of this approach lies in the crucial role of Aβ in AD pathogenesis. While there is criticism of the “amyloid hypothesis” of disease development, the role of this protein in pathology remains undeniable [15]. Evidence suggests that Aβ aggregation is an essential factor in the pathological cascade initiation leading to protein aggregation, neuroinflammation, and tau phosphorylation [16,17]. Along with this, the formation of senile plaques of Aβ as well as its content in the cerebrospinal fluid (CSF) are important AD markers.

Senile plaques containing Aβ found in patients’ brain tissue have been shown to consist of various modified forms of the protein, some of which may have a high degree of pathogenicity [18,19]. One such modification is isomerization of aspartic acid residue (Asp) 7 [20,21], which accounts for up to 50% of total detectable amyloid protein in plaques [22]. The amount of this isomerized form increases with age and accumulates in the brain tissue of patients with AD [23]. Previous studies have shown that Asp7 isomers are highly toxic, affecting neuronal cell cultures, inducing amyloidogenesis in vivo in transgenic mice, and leading to tau phosphorylation [24,25,26,27], suggesting its importance in AD progression. This makes Asp7 a promising target for translational research, including as a potential agent for injectable disease models.

Metal ions, primarily zinc ions, play a crucial role in the toxic effects of amyloid proteins, influencing protein aggregation [28,29,30]. Recent evidence suggests that the metal-binding site (1–16) of the Asp7 isoform of Aβ shows a higher affinity for zinc-induced oligomerization, which may influence the aggregation and neurotoxic properties of the full-length form of the peptide [31]. Understanding the structural features and aggregation properties of these proteins is essential for developing therapeutic approaches [32,33], making it a critical task to study these aspects of Asp7.

Therefore, our study aimed to compare the morphological and aggregation characteristics of synthetic Aβ_42_ with its Asp7 isomer and analyze the effects of their intracerebroventricular (i.c.v.) administration on cognitive function and oxidative stress in rats.

## 2. Materials and Methods

**Figure 1:**
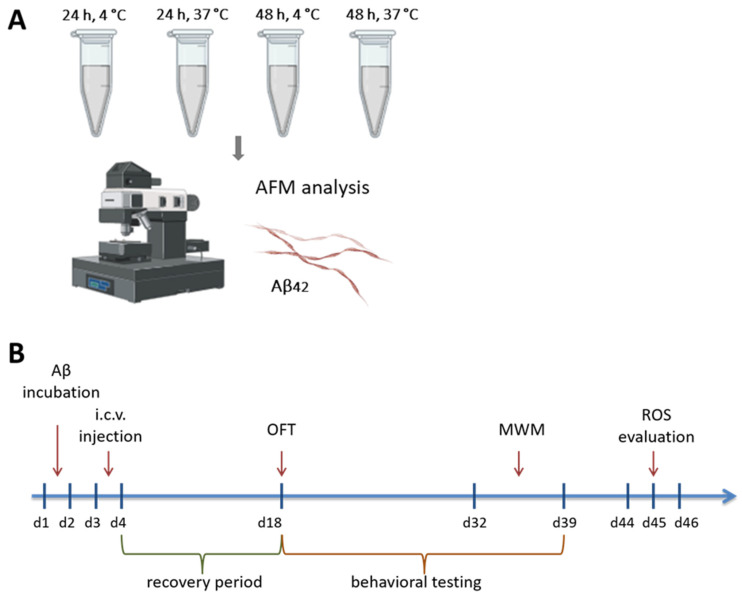
Scheme of the experiment. (**A**) Stage 1. Evaluation of morphological and aggregation properties of Aβ_42_ and Asp7 iso-Aβ_42_ under various incubation conditions using AFM. (**B**) Stage 2. Scheme of the experimental procedure after i.c.v. administration of Aβ_42_ and Asp7 iso-Aβ_42_. AFM—atomic force microscopy, i.c.v.—intacerebroventricular, OFT—open field test, MWM—Morris water-maze test, ROS—reactive oxygen species.

The work was divided into two stages. In the first stage, we investigated the morphology and aggregation characteristics of Aβ_42_ solutions and their Asp7 isoform using atomic force microscopy (AFM) (NT-MDT SI, Zelenograd, Russia). Solutions were analyzed under different temperature conditions and incubation periods: 24 h at 4 °C, 24 h at 37 °C, and 48 h at both temperatures. A schematic illustration of the experimental setup is provided in Figure 1A.

In the second phase of the study, we selected the appropriate incubation conditions for the production of Aβ_42_ and Asp7 iso-Aβ_42_ oligomers based on the AFM data (24 h at 4 °C). The resulting solutions were administered stereotactically to laboratory rats. The effect of these preparations on the cognitive abilities of the animals and the level of oxidative stress in brain tissue was then analyzed. A schematic representation of the second phase of this study is provided in Figure 1B.

### 2.1. Preparation of Synthetic Aβ42 and Asp7 Iso-Aβ42

Synthetic peptides Aβ_42_ ([H2N]-DAEFRHD SGYEVHHQKLVFFAEDVGSNKGAIIGLMVGGVVIA-[COOH]) and Asp7 iso-Aβ_42_ ([H2N]-DAEFRH[isoD]SGYEVHHQKLVFFAEDVGSNK GAIIGLMVGGVVIA-[COOH]) were purchased from Biopeptide (San Diego, CA, USA). Peptides were dissolved in 1 mL 25% NH3 and aliquoted to 200 µL (0.2 mg). After a 15 min incubation period, solutions were liophylizated and stored at −20 °C.

For the AFM experiments and further i.c.v. administration, monomers were dissolved in the artificial CSF (aCSF) (127 mM NaCl, 1.0 mM KCl, 1.2 mM KH_2_PO_4_, 26 mM NaHCO_3_, 10 mM D-glucose, 2.4 mM CaCl_2_, 1.3 mM MgCl_2_) and incubated according to the selected protocol.

### 2.2. AFM

The following protocols of incubation were used to analyze the structure and aggregation of Aβ42 and Asp7 iso-Aβ42: 24 h, 4 °C; 24 h, 37 °C; 48 h, 4 °C; 48 h, 37 °C.

For AFM scans, 10 μL of each sample was placed on freshly cleaved mica and incubated in a Petri dish for 10 min for peptide adsorption onto the mica. After adsorption, the mica samples were rinsed with 1 mL of ultrapure water and dried in an argon stream.

AFM measurements were conducted using an NTEGRA II microscope (NT-MDT SI, Zelenograd, Russia) in semi-contact mode with silicon cantilevers (spring constant of 1.74 N/m, 90 kHz). The scanning rate was typically 0.5 Hz.

AFM images were processed using Femtoscan software (version 2.4.10) (Advanced Technologies Center, Moscow, Russia) [34]. No further image modification except the line alignment along the fast scan direction and the surface parabolic subtraction was applied. For the statistical analysis, the oligomeric aggregates were measured by their diameters, which were traced from the images by circular segments using the special tool in the software (Figure S1). The total number of measured oligomeric aggregates for each sample from the corresponding images was as follows: N(Aβ42) = 702, N(iso-Aβ42) = 630. The selected contours were converted into sequences of two-dimensional coordinates, and the obtained data were presented in the form of box plots and histograms with bin size five with one-way ANOVA analysis. For fibrillar structures, their lengths were traced manually using the same software.

### 2.3. Experimental Animals

The study used male Wistar rats (n = 26) from the Nursery for Laboratory Animals (Pushchino, RAS, Moscow Region). The rats were approximately 2.5 months old and weighed 200–250 g. The animals were kept at 20–22 °C, in non-inverted 12 h daylight hours, with access to food and water ad libitum. All procedures involving the animals were conducted in accordance with relevant European and Russian guidelines for animal research and approved by the local ethical committee of Serbsky National Medical Research Center of Psychiatry and Narcology of the Ministry of Health of Russia (protocol №2 from 19.10.2023).

During the study, the rats were divided into three groups: a control group (n = 9) that underwent sham surgery, a group that received an i.c.v. injection of a solution of Aβ42 oligomers (n = 8), and a group that also received an i.c.v. injection, but with Asp7 iso-Aβ42 oligomers (n = 9).

### 2.4. Surgical Operation

For i.c.v. injection, peptides Aβ42 and Asp7 iso-Aβ_42_ were incubated in aCSF for 24 h at 4 °C to obtain an oligomer form.

For surgery, the animals were anesthetized prior to surgery by intraperitoneally (i.p.) injecting a 1:1 mixture of ketamine (50 mg/kg) (Endopharm, Moscow, Russia) and diazepam (125 mg/kg) solutions (Polfa Warszawa, Warsaw, Poland). The i.c.v. injection of the pre-prepared solution of Aβ_42_ oligomers and Asp7 iso-Aβ_42_ isoform was performed bilaterally according to the following coordinates from the rat brain atlas: (AP: −1; ML: ± 1.5; DV: −4.3). Brain stereotaxic apparatus (RWD Life Science, Shenzhen, China) was used to determine the insertion coordinates. To insert the injection needle, a small incision was made in the skin and muscles on top of the head of the animal. The skull surface was then cleaned with 70% ethanol solution and hydrogen peroxide to remove any blood residue. Using a drill (RWD, Shenzhen, China), a hole was made bilaterally in the skull where the injection needle could be inserted. The volume of fluid injected into one hole was 10 µL. The injection was performed using a Hamilton syringe mounted on an automatic injector (RWD Life Science, Shenzhen, China) at a rate of 2 µL/min. Within 5 min after substance injection, the needle remained inside the brain tissue to prevent the fluid from entering other brain structures.

The sham-operated control animals were stereotactically injected with aCSF fluid solution in an identical volume. After the needle removal, the tissues were disinfected with a chlorhexidine solution (0.05%) and sutured using self-absorbable suture material Vicryl (Ethicon, Raritan, NJ, USA).

### 2.5. Behavioral Testing

Behavioral testing was conducted between 10:00 a.m. and 2:00 p.m., and animals were transferred to the testing room at least 30 min prior to the start of the test. Animal behavior was recorded via a digital video camera and analyzed using the “RealTimer” program (Open Science, Moscow, Russia).

#### 2.5.1. Open Field Test (OFT)

The test was conducted in a 45 × 45 × 45 cm arena made of gray plastic, illuminated by a 200 watt lamp. The animal was placed in the center of the arena, and movement was recorded using a digital video camera located at a distance of 180 cm from the arena. Motor activity in the arena was measured automatically using laser detection via a Multi-Conditioning device (TSE System, Berlin, Germany). The duration of the distance traveled and the number of rearing behaviors were measured.

#### 2.5.2. Morris Water Maze Test (MWM)

The Morris maze was a pool with gray walls, 150 cm in diameter and 60 cm high, filled with water at 40 cm. The water temperature was 24 °C. An 8 cm diameter circular platform made of clear plastic was placed in the center of one of the quadrants of the pool 2 cm below the surface of the water. The pool was located in a room with a large number of ambient spatial landmarks.

The animals were trained over one day, during which they were placed in the water from eight different starting points. After the animal reached the platform, it was left on it for 15 s, then placed in a separate cage for 60 s. Rats that did not reach the platform during 60 s were gently guided to it. In each trial, the time required to reach the platform was recorded. After 48 h, spatial memory was assessed in a single test trial where the time to reach the platform (s) was recorded. One week later, the platform was removed from the pool, and the time spent by the rat in the quadrant where the platform was previously located was recorded. Throughout the experiment, environmental stimuli and platform location remained consistent.

### 2.6. Intravital Evaluation of Oxidative Stress in Rat Brain Tissue

#### 2.6.1. Instrumentation

All electrochemical measurements were carried out at room temperature using a two-electrode configuration with an AgCl electrode as the counter-reference electrode (a 0.3 mm AgCl coated Ag wire); all potentials were reported vs Ag/AgCl reference. In vivo voltammetric experiments were performed at room temperature (24 ± 2 °C) inside a Faraday cage. The Faradaic current was measured with a MultiClamp 700B patch-clamp amplifier (Molecular Devices, San Jose, CA, USA). Transfer and recording of measurements to a computer was carried out using the ADC–DAC converter Axon Digidata 1440B (Molecular Devices, San Jose, CA, USA) and the software pClamp version 10. The micromanipulator PatchStar (Scientifica, Uckfield, UK) was used to feed the nanosensor. All manipulations were made on the table of an optical inverted microscope (Nikon, Tokio, Japan). The current signals were filtered with 0.5 kHz lowpass filters.

#### 2.6.2. Nanoelectrodes Fabrication

Platinum nanoelectrodes (PtNEs) were prepared based on commercially available disk-shaped carbon nanoelectrodes (CNE) isolated in quartz (ICAPPIC Limited, London, UK) with diameters of 50–150 nm. Preparation of CNE has been described in detail previously [35,36,37]. Briefly, a platinum electrode is a nanopipette filled with carbon. The CNEs were initially placed in 1 mM ferrocene methanol in buffered saline solution (PBS) to verify their operability for further work. Electrochemical etching was performed by means of cyclic voltammetry (CV) from 0 to 2.2 V in 0.1 M KOH, 10 mM KCl for typically 15–40 cycles until the formation of nanocavities. Then platinum was deposited to increase the electrochemical activity of the surface. Electrochemical deposition of platinum was achieved by cycling from 0 to −0.8 V with a scan rate of 200 mV s^−1^ for 4−5 cycles in 2 mM H_2_PtCl_6_ solution in 0.1 M hydrochloric acid. The diameter of the PtNE was approximately 50−150 nm, and showed excellent electrochemical performance. The electrocatalytic sensitivity of the Pt-black tip and its selectivity for reactive oxygen species (ROS)/nitrogen species (RNS) allowed us to measure ROS/RNS generation. [38]. This nanoelectrode was sensitive to ROS, as has been shown in previous studies [36,39]. We investigated the reproducibility of the nanoelectrode fabrication by preparing several nanoelectrodes (N = 5) with identical pulling parameters, depositing carbon, and averaging their steady state.

#### 2.6.3. In Vivo Electrochemical ROS Measurements

Before the measurement, the rats were anesthetized prior to surgery by i.p. injecting a 1:1 mixture of ketamine (50 mg/kg) (Endopharm, Moscow, Russia) and diazepam (125 mg/kg) solutions (Polfa Warszawa, Warszawa, Poland). Brain stereotaxic apparatus (RWD, Shenzhen, China) was used to determine the insertion coordinates. To insert the nanoelectrodes, a small incision was made in the skin and muscles on top of the head of the animal. The skull surface was then cleaned with 70% ethanol solution and hydrogen peroxide to remove any blood residue. Using a drill (RWD Life Science, Shenzhen, China), a hole was made in the skull according to the selected coordinates.

Next, the rat in stereotaxis was placed on an antivibration table. The hole was approached using a micromanipulator (Scientifica, Uckfield, UK), which was mounted on a stand above the rat’s head. The electrode was inserted strictly vertically. The reference electrode was placed in the folds of the skin to ensure contact with body fluids. Measurements were carried out in pre-drilled holes (cortex: AP: +3, ML: −4, DV: −1, hippocampus: AP: −4, ML: −4, DV: −3.5).

The cyclic voltammetry (CV) had been previously measured in PBS (sweep rate 400 mV/s, from −800 mV to +800 mV). Next, using a micromanipulator, the electrode was brought to the selected hole and the electrode was lowered to depths of 1000 µm (AP: +3, ML: −4, DV: −1) и 3500 µm (AP: −4, ML: −4, DV: −3.5) in steps of 100 µm. Similar CV measurements were recorded (scan rate 400 mV/s, from −800 mV to +800 mV). The overall ROS measurement was carried out at a potential of +800 mV. Next, in order to avoid effects associated with the heterogeneity of the biological environment, normalization was carried out to the ROS level in the control. Since the size of nanoelectrodes can vary from electrode to electrode, this internal normalization is necessary for all measurements.

### 2.7. Statistical Processing

Statistical analysis of the obtained data and graphing were performed using RStudio software (Version 1.4, RStudio PBC, Boston, MA, USA) and jamovi (Version 1.6, The jamovi project, Sydney, Australia).

After obtaining the data, the Shapiro–Wilk test was used to check for normality. Based on this, the choice of statistical criteria for comparison was made: parametric or non-parametric. In the case of normal distribution, Mean ± SEM was considered; in the case of non-normal distribution, Med (Q1; Q3) was considered.

Parametric tests (for normally distributed data) included analysis of variance (ANOVA) followed by Tukey’s post hoc test. Non-parametric tests (for non-normally distributed data) involved the Kruskal–Wallis rank sum test followed by the Dwass–Steel–Critchlow–Fligner post hoc test. Differences were considered significant at *p* < 0.05. 

## 3. Results

Our study was the first to investigate the effects of i.c.v. injection of the Asp7 iso-Aβ_42_ on the development of AD signs in laboratory rats and evaluate the structural and aggregation properties of the protein using AFM. Specifically, we chose to administer the oligomeric form of Aβ_42_ in order to enhance its potential neurotoxic effects. In order to validate the appropriate level of oligomerization and assess differences in fibrillation processes between isomerized and non-isomerized Aβ_42_, we evaluated the degree of aggregation in aCSF solutions at various incubation times and temperatures. This initial stage of our work also involved selecting an optimal incubation protocol.

### 3.1. Comparison of Morphologic and Aggregation Properties of Aβ42 and Asp7 Iso-Aβ42 and Selection of the Optimal Incubation Protocol

**Figure 2:**
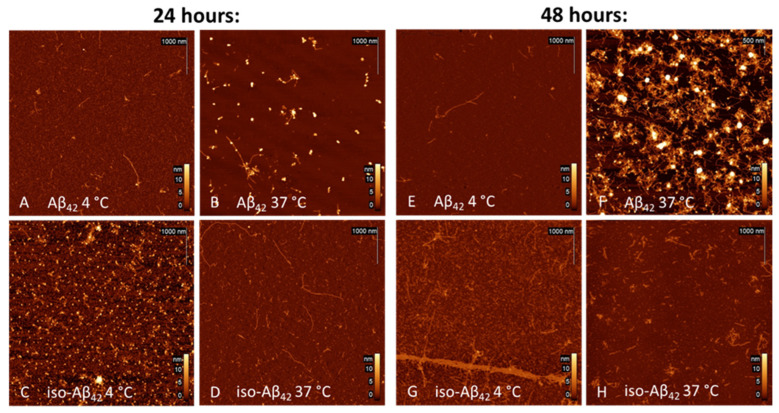
Aβ aggregates imaged via AFM. (**A**) Aβ_42_, incubated for 24 h at 4 °C; (**B**) Aβ_42_, incubated for 24 h at 37 °C; (**C**) iso-Aβ_42_, incubated for 24 h at 4 °C; (**D**) iso-Aβ42, incubated for 24 h at 37 °C; (**E**) Aβ_42_, incubated for 48 h at 4 °C; (**F**) Aβ_42_, incubated for 48 h at 37 °C; (**G**) iso-Aβ_42_, incubated for 48 h at 4 °C; (**H**) iso-Aβ_42_, incubated for 48 h at 37 °C.

We performed an AFM analysis of incubated Aβ_42_ and its Asp7 isoform aggregates (Figure 2) to compare its aggregation properties and to select an optimal oligomer incubation protocol. Aggregation of peptides of Aβ_42_ and its Asp7 isoform was performed for 24 and 48 h at 4 and 37 °C.

Incubation of Aβ_42_ for 24 h at 4 °C leads to the formation of oligomeric structures (Figure 2A)—their content in relation to fibrillar forms predominates. An increase in the temperature of incubation to the physiological 37 °C affects the morphology of the aggregates—the predominance of clearly defined fibrils with lengths up to 1500 nm (Figure S1A) is observed, and their structure appears rigid (Figure 2B). Incubation of iso-Aβ_42_ at different temperature conditions also leads to the formation of different forms of aggregates. At 4 °C, iso-Aβ_42_ forms oligomeric structures that differ in shape and size from Aβ_42_ oligomers (Figure 2C). At incubation at 37 °C, iso-Aβ_42_ forms predominantly fibrillar structures (Figure 2D) with lengths up to 1870 nm (Figure S1B) with a more bent and twisted shape. After prolonged incubation for 48 h at 4 °C, in the Aβ_42_ sample no significant changes are observed (Figure 2E), and in the case of the iso-Aβ_42_ sample, fibrillar structures begin to predominate (Figure 2G). With continued incubation at 37 °C, the appearance of fibrillar structures changes dramatically: Aβ_42_ aggregation accelerates, and fibrils form a dense plaque-like network (Figure 2F); alternatively, the iso-Aβ_42_ forms “soft” structures similar to protofibrils (Figure 2H), their length is visually reduced, but their shape becomes wider.

**Figure 3:**
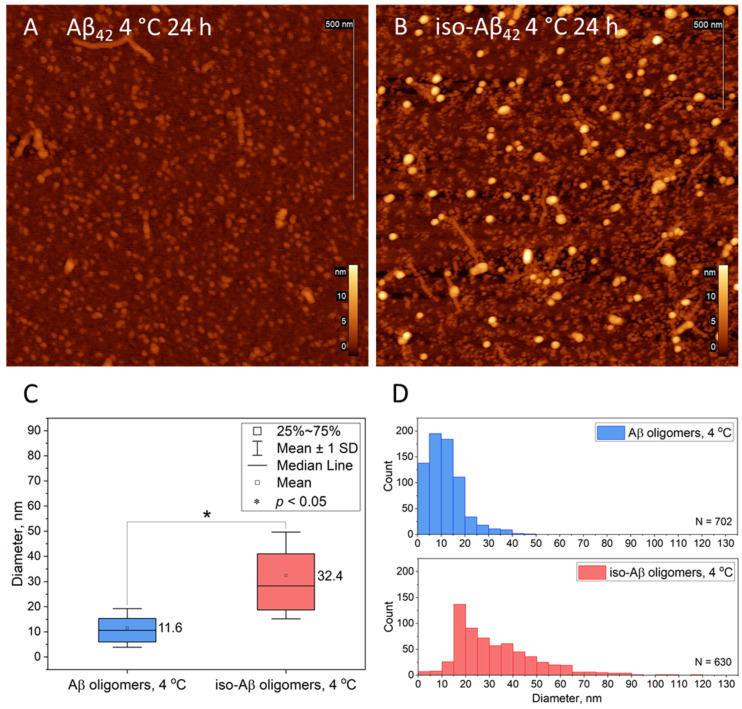
Oligomeric aggregates (4 °C, 24 h), imaged via AFM, with statistical analysis: (**A**) Aβ oligomers; (**B**) iso-Aβ oligomers; (**C**) box plots of two diameter arrays (* *p* < 0.05, one-way ANOVA); (**D**) histograms of oligomer diameters (N(Aβ_42_) = 702, N(iso-Aβ_42_) = 630).

Based on the images presented, it can be concluded that the temperature and incubation time have a significant impact on the level and pattern of protein aggregation. The optimal conditions for obtaining oligomeric forms of both Aβ_42_ and its isoform appear to be incubation for 24 h and 4 °C. Statistical analysis of oligomeric aggregates was performed using FemtoScan software (version 2.4.10)on obtained AFM images and is presented in Figure 3. The size of the aggregates was measured by their diameter (examples are shown in Figure S1C–E, marked with red circles; fibrillar aggregates were excluded from the analysis), histograms of the diameter distribution were constructed on the basis of the obtained arrays, and one-way ANOVA analysis was performed.

As can be seen from the graph in Figure 3C, the sizes of the obtained oligomers are statistically significantly different. Thus, the diameter of Asp7 amyloid isoform oligomers exceeds the diameter of Aβ_42_ oligomers by almost three times (*p* < 0.05). The distribution graph of different forms of peptides after incubation for 24 h at 4 °C also reflects the differences in size and morphology (Figure 3D). Thus, we can see that Aβ_42_ oligomers are represented mainly by structures with diameters from 5 to 10 nm and from 10 to 15 nm, with a number of very small formations with diameters up to 5 nm (Figure 3D, blue bars). In the Asp7 iso-Aβ_42_ solution, the main mass of particles is represented by oligomers with a diameter of 15–20 nm, although a number of larger aggregates of more than 20 nm and a small number of protofibrils are also observed (Figure 3D). The data obtained indicate that oligomers of Aβ_42_ and its Asp7 isoform differ significantly morphologically, which may mediate different effects of stereotactic administration. The average diameter of amyloid oligomers was 11.6 nm (Figure 3C, blue box), the range of data is relatively small (Figure 3D, blue bars), which may indicate the uniformity of the oligomers’ shape (excluding fibrillar forms). Amyloid isoforms have a larger diameter, an average value of 32.4 nm (Figure 3D, red box), and a relatively large range of data (Figure 3C, red bars).

### 3.2. Effect of i.c.v. Administration of Aβ_42_ and Asp7 Iso-Aβ_42_ on the Behavior of Laboratory Animals

In view of the AFM data obtained, a period of 24 h at 4 °C was selected as the optimal incubation protocol for obtaining the oligomeric form of the protein.

**Figure 4:**
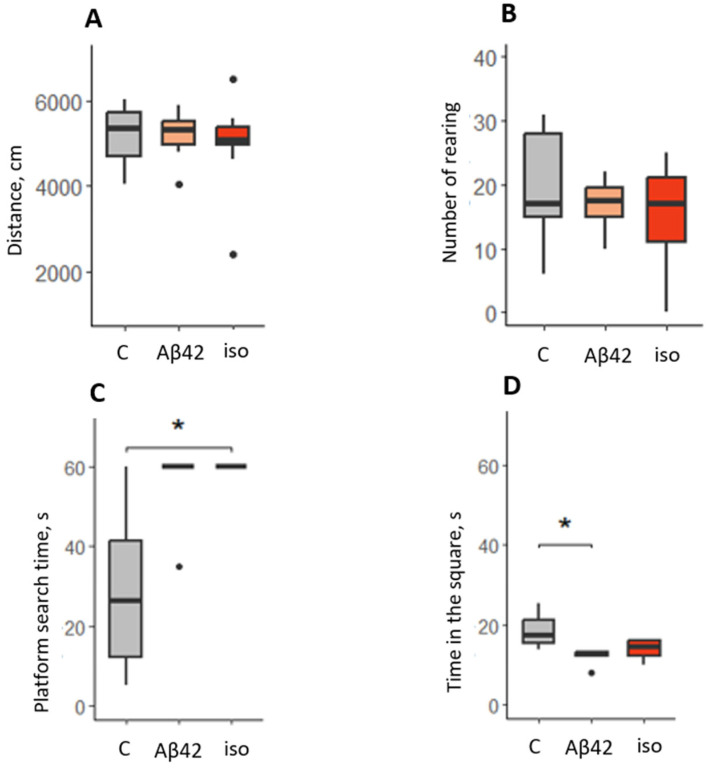
Behavioral assessment after i.c.v. Aβ injection. (**A**) distance traveled (cm) in the OFT; (**B**) number of rearing behaviors in the OFT; (**C**) platform search time (s) in the MWM test (test trial, 48 h); (**D**) time in the square where platform was earlier located (s) in the MWM test (test trial, 1 week). C—sham-operated control group, Aβ_42_—Aβ_42_ injected group, iso—Asp7 iso-Aβ_42_ injected group. *—*p* < 0.05.

First of all, the level of locomotor activity of animals and exploratory behavior in the OFT test was evaluated (Figure 4A,B).

The test results showed that the level of locomotor and exploratory activity did not differ between the groups (χ^2^ = 0.21, *p* = 0.9 and F = 0.63, *p* = 0.547, respectively). Thus, the median values of the measures of distance traveled and the number of rearing behaviors were similar (Figure 4A,B).

However, in the MWM test, aimed at assessing spatial memory and performed at a later time after surgery, differences between groups were noticeable (Figure 4D. Specifically, in the test trial 48 h after training, there were significant differences between the control and experimental group (χ^2^ = 9.548, *p* = 0.008). The duration of platform searching was significantly longer for the iso group compared to the control (*p* < 0.05). Despite the lack of significant differences, the Aβ_42_ group showed a pronounced tendency to increase the index (*p* = 0.055). In general, it should be noted that animals of the experimental groups practically did not reach the platform. The learning ability of the rats also differed between the groups: during training, approximately 47.6% of animals in the control group trained successfully, while only 16.7% in the amyloid group and 25.7% in the Asp7 iso-Aβ_42_ group were successfully trained. The number of successfully trained animals differed significantly between the experimental groups and the control by the χ^2^ criterion (*p* < 0.05). In the test one week later, when the platform was removed from the experimental setup, the values of time spent in the square where it was previously located were also significantly different between the groups (χ^2^ = 8.148, *p* = 0.017). A significant decrease in time was shown for the amyloid group compared to controls (*p* < 0.05). Thus, the data obtained indirectly indicate the development of pathologic symptoms at later postoperative time.

### 3.3. Effect of i.c.v. Administration of Aβ_42_ and Asp7 Iso-Aβ_42_ on the Level of Oxidative Stress in the Brain of Experimental Animals

**Figure 5:**
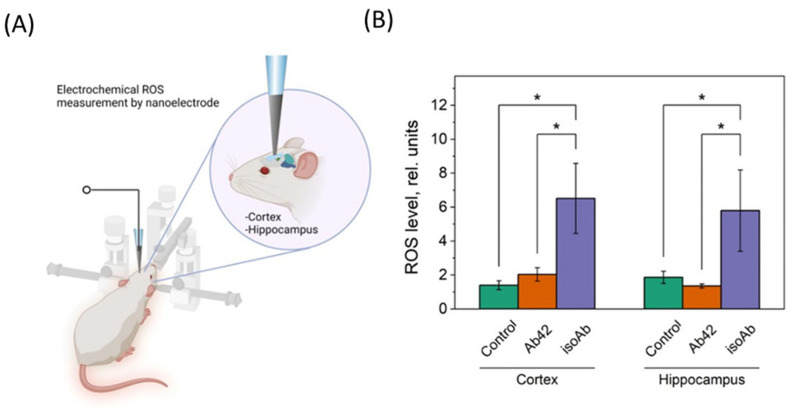
In vivo ROS electrochemical measurement in rat brain. (**A**) Scheme of ROS measurement in cortex and hippocampus by Pt nanoelectrode; (**B**) ROS level in rat brain (cortex, hippocampus) after injection of Aβ_42_ or Asp7 iso-Aβ_42_. C—sham-operated control group, Ab_42_—Aβ_42_ injected group, isoAb—Asp7 iso-Aβ_42_ injected group. *—*p*< 0.05.

In the context of AD, Aβ plaques induce excessive production of ROS, exacerbating neuronal damage. Aβ misfolding generates neurotoxic aggregates that increase ROS levels, resulting in progressive oxidative stress and cell death. This dual burden of ROS production and Aβ aggregation creates a vicious cycle where oxidative damage promotes further Aβ production, worsening AD symptoms [40]. ROS measurements in the rat brain were performed in the cerebral cortex and hippocampus after i.c.v. injection of Aβ_42_ and its Asp7 isoform using an electrochemical approach (Figure 5A,B).

For the experiment, three groups of animals (N = 5) were selected. The first group comprised control rats, the second group of rats was injected with Aβ_42_ (10 µL, 500 µg/mL) into the cerebral ventricles, the third group of rats was injected with the Asp7 Aβ_42_ isoform (10 µL, 500 µg/mL). ROS measurements were performed on day 44–46 after administration of the Aβ_42_ and Aβ_42_ isoform (Figure 5A). It has been shown that when iso-Aβ_42_ is administered, there is a significant increase in ROS levels within the hippocampus and cerebral cortex (*p* < 0.05) (Figure 5B). An insignificant increase of ROS level in the cerebral cortex was also observed in the Aβ_42_ group (Figure 5B).

## 4. Discussion

In view of the high prevalence of AD and the lack of effective treatments for the disease, identifying the factors involved in its development through translational research presents a significant task. In this study, we compared the morphological and aggregation properties of amyloid peptides—Aβ_42_ and its Asp7 isoform—and analyzed the effects of i.c.v. administration of oligomeric forms of the peptides.

Comparison of the properties of the peptides at different incubation times using AFM revealed differences in the sizes of oligomers and fibrils formed. In addition, Aβ42 and its isoform also differed in the intensity of aggregate formation. The AFM technique has been demonstrated to be well-suited for characterizing amyloidogenic peptides and proteins with aggregative properties, as it allows for obtaining high-resolution morphological images without the need for extensive chemical manipulation [41,42], and allows for the selection of optimal incubation protocols for achieving the desired aggregative form [41].

Analysis of data obtained from shorter incubation periods (24 h) at lower temperatures (4 °C) revealed an abundance of the oligomeric form of peptides in the samples studied. Conversely, more intensive peptide aggregation and fibril formation were observed during the same incubation at 37 °C. Fibril accumulation was particularly notable at higher temperatures and longer incubation times (48 h). Data from AFM studies indicate the appearance of oligomers almost immediately after the start of peptide incubation at room temperature and the formation of short fibrils already at the stage of several hours after the start of incubation [43,44]. At 37 °C, active aggregation occurs a couple of hours after the start of incubation [45]. It has been shown that temperature, pH, and other incubation conditions significantly affect the protein aggregation process [46,47]. In particular, low temperature conditions, on the contrary, can slow down the process [48]. Some researchers suggest that exposure to reduced temperatures in conjunction with low salt concentrations can stabilize oligomeric forms [49]. These findings are consistent with results from animal models using exactly the 24 h and 4 °C protocol to produce Aβ42 oligomers [50,51]. Oligomers are believed to be highly toxic, mediating numerous pathogenic properties and playing a crucial role in AD pathogenesis [52,53,54].

Interestingly, the Aβ_42_ and Asp7 iso-Aβ_42_ oligomers exhibited significant differences in size. The median particle size of the amyloid isoform was approximately three times larger than that of the unmodified peptide, and the particle size distribution also shifted towards larger formations. Furthermore, the intensity and shape of fibril filaments formed at later time points differed as well. Mastrangelo et al. in their analysis of amyloid aggregation at early time points distinguished between oligomers of the lowest molecular weight (cross-sectional dimensions of approximately 5–10 nm), high molecular weight oligomers (cross-sectional dimensions of approximately 15–25 nm) of larger size, and protofibrils (longer than 40 nm), the formation of which is influenced by incubation time and concentration [43]. It can be hypothesized that incubation of synthetic Aβ_42_ results in the formation of largely low molecular weight oligomeric forms, whereas incubation of the isoform results in larger oligomers and protofibrils. The structural properties of Aβ proteins can significantly influence their toxicity, in view of which, the demonstrated neurotoxic effects of the Asp7 isoform may be mediated, among others, by its morphology [55]. In particular, structure and steric properties may also affect the binding of peptides to metal ions, including zinc [56,57]. Zirah et al. showed a higher affinity of the isoform for zinc [58]. Perhaps the structural features identified in the experiment also influence this process. It is worth noting that the high content of oligomeric forms persisted for a longer period specifically for the Asp7 sample. Previous studies on complete amyloid isomers showed preservation of oligomers for up to 23–25 h [59]. It can be assumed that this is also due to the higher toxicity of the isoform shown in the literature [60].

Despite the pronounced morphological differences between Aβ_42_ and Asp7 iso-Aβ42 oligomers, behavioral analysis revealed no significant differences in the effects of i.c.v. injection of peptides on cognitive impairment. The administration of the drugs had no impact on the locomotor and exploratory activity of the animals in the OFT, consistent with the findings of some studies [61,62]. Some evidence suggests that cognitive impairment may manifest or worsen later after stereotactic amyloid injection [63,64,65]. Indeed, behavioral testing later after surgery showed marked memory impairments in the MWM test after administration of Aβ_42_ and Asp7 iso-Aβ_42_: in the learning phase, during the test, and during the assessment of long-term memory. The development of AD is accompanied by impairments in spatial memory and spatial orientation [66]. In view of this, a number of translational studies demonstrate the presence of cognitive dysfunction in the MWM, which is one of the main methods for assessing spatial memory in rodents [67,68,69].

Although behavioral analysis did not reveal any significant differences in toxicity between conventional and isomeric forms of the protein, intravital analysis of oxidative stress using PtNEs revealed an increase in ROS in the hippocampus and cortex of rats treated with the Asp7 iso-Aβ_42_. Increased oxidative stress is a hallmark of neurodegenerative pathologies and is closely associated with synapse damage, aging, and neuroinflammatory processes [70,71]. ROS generation and mitochondrial dysfunction are the main reasons for the multifaced toxicity observed in neuronal cells during incubation with Aβ aggregates [72]. Primary ROS and RNS can be directly oxidized or reduced using fixed electrochemical potentials. Therefore, ROS/RNS can be detected and quantified using electroanalytical methods. Electrochemical methods have proven to be very useful for in vivo quantitation of analytes due to their several advantages. Previously, it has been shown that electrochemical nanoelectrodes are able to quantify total ROS/RNS levels in single cells and tumor tissues [36,37].

Recent studies of the Asp7 isoform of Aβ have shown that the peptide induces mitochondrial damage, and ROS and RNS accumulation, causing significant oxidative stress in vitro [73]. These data may indicate that the mechanism of neurotoxic effects of the Asp7 Aβ isomer may be directly related to oxidative stress.

Thus, our results revealed morphologic and aggregation differences between Aβ_42_ and Asp7 iso-Aβ_42_, and demonstrated pathogenic effects of the isomeric form on cognitive function and oxidative stress levels in vivo. These data indicate the potential of Asp7 iso-Aβ_42_ for AD modeling studies in rodents. However, a number of additional studies are required to establish and validate the pathology model, including detailed analysis of the effects of the isomer on behavior and neuroinflammatory processes, as well as amyloid accumulation in brain tissue. We hypothesize that our work could be the first step towards evaluating and developing a model of neurodegeneration based on this isomer.

Our study has some limitations. One of them is the time period before the evaluation of cognitive function. Typically, a rather short duration of postoperative recovery time is used. However, due to the fact that some studies have indicated a decline in cognitive abilities in animals at a later point in time, and considering that we did not find any differences in exploratory behavior two weeks after surgery, we decided to conduct memory measurements two weeks after the evaluation of motor activity. The second limitation of our research is the invasive nature of ROS measurement procedures and the necessity for repetition of stereotactic procedures. Despite this, the surgical intervention did not significantly affect the health condition of the animals, making it a convenient tool for long-term oxidative stress assessment.

## 5. Conclusions

In summary, this study highlights the distinct morphological and aggregation properties of Aβ_42_ and its Asp7 isoform, contributing to a better understanding of their role in AD pathogenesis. The use of AFM revealed significant differences in oligomer and fibril formation between the two peptides, with the Asp7 isoform resulting in larger and more persistent oligomeric structures, which may contribute to its increased neurotoxicity. These structural variations suggest that the Asp7 isoform may play a crucial role in oxidative stress, a key factor in neurodegeneration, as demonstrated by the elevated ROS levels observed in the hippocampus and cortex of rats treated with the isoform, and may induce cognitive impairments, as revealed by spatial memory evaluation.

## Data Availability

The data presented in this study are available on request from the corresponding author due to the absence of special open data repositories and the storage of data by the authors of the study.

## Supplementary Materials

The following supporting information can be downloaded at: https://www.mdpi.com/article/10.3390/brainsci14101042/s1, Figure S1: Demonstration of fibril lengths (A, B, dashed lines with dots) and measurement of oligomers (C, D, and E, red circles) using FemtoScan software for the AFM images for statistical data. For iso-Aβ 4 °C, statistical analysis was carried out for two types of objects (D, E), and the obtained data were combined.
