# Supplementary material for: Beta-Amyloid and Its Asp7 Isoform: Morphological and Aggregation Properties and Effects of Intracerebroventricular Administration
Source: Brain Sci. 2024 Oct 21;14(10):1042. doi: 10.3390/brainsci14101042 (PMC11506273; doi:10.3390/brainsci14101042)
Supplement: Supplementary file 1 [file brainsci-14-01042-s001.zip › brainsci-3258842-supplementary.pdf]

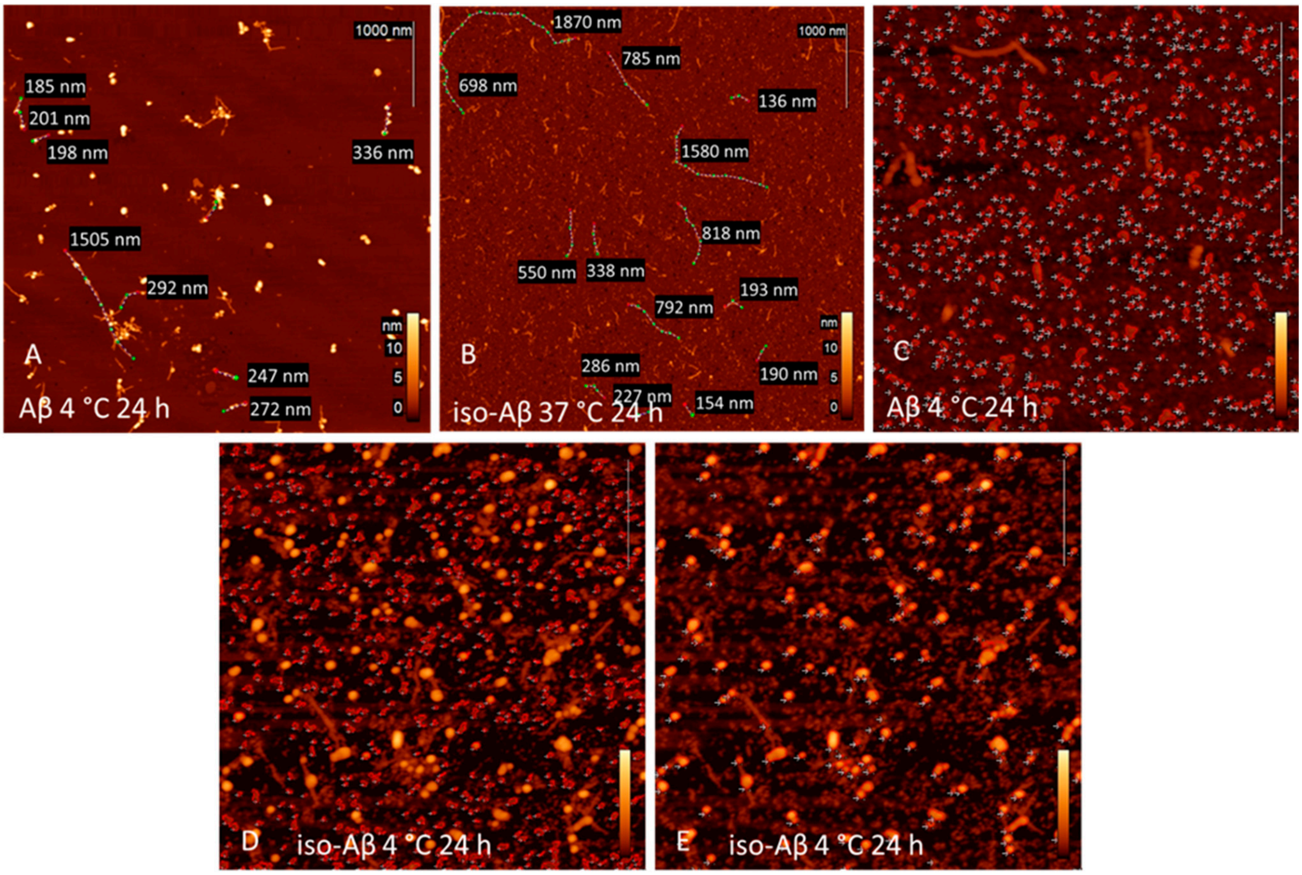

Figure S1 – Demonstration of fibrils lengths (A, B, dashed lines with dots) and measurement of oligomers (C, D and E, red circles) in FemtoScan software for the AFM images for statistical data. For iso-amyloid 4 °C, statistical analysis was carried out for two types of objects (D, E), the obtained data were combined
